# Biometric Identity Based on Intra-Body Communication Channel Characteristics and Machine Learning

**DOI:** 10.3390/s20051421

**Published:** 2020-03-05

**Authors:** Ahmed E. Khorshid, Ibrahim N. Alquaydheb, Fadi Kurdahi, Roger Piqueras Jover, Ahmed Eltawil

**Affiliations:** 1 Electrical Engineering and Computer Science Department, University of California, Irvine, CA 92697, USA; alquaydi@uci.edu (I.N.A.); kurdahi@uci.edu (F.K.); ahmed.eltawil@kaust.edu.sa (A.E.); 2 Bloomberg LP, New York, NY 10022, USA; rpiquerasjov@bloomberg.net; 3 Computer, Electrical and Mathematical Science and Engineering Division (CEMSE), King Abdullah University of Science and Technology, Thuwal 23955, Saudi Arabia

**Keywords:** body area networks, channel gain/attenuation, channel modeling, galvanic coupling, intra-body communications, phantoms, tissue mimicking materials, ultralow power systems

## Abstract

In this paper, we propose and validate using the Intra-body communications channel as a biometric identity. Combining experimental measurements collected from five subjects and two multi-layer tissue mimicking materials’ phantoms, different machine learning algorithms were used and compared to test and validate using the channel characteristics and features as a biometric identity for subject identification. An accuracy of 98.5% was achieved, together with a precision and recall of 0.984 and 0.984, respectively, when testing the models against subject identification over results collected from the total samples. Using a simple and portable setup, this work shows the feasibility, reliability, and accuracy of the proposed biometric identity, which allows for continuous identification and verification.

## 1. Introduction

Wearable sensors and systems are rapidly being adopted as means of augmenting and improving health care services. In order to provide a cable-free biomedical monitoring system, new wireless technologies associated with sensor applications have been promoted as the next biomedical revolution, promising a significant improvement in the quality of health-care applications. Yet the size and power requirements of wireless sensors that are typically dominated by the RF section of the associated transceivers, have limited their adoption, leading to a rise in Intra-Body Communication (IBC) systems where data transmission is carried out through the body (mostly skin layers), rather than through air [1,2]. Radio Frequency (RF) front ends, for each individual node are replaced with simpler interfaces resulting in lower power and reduced area.

The first challenge facing IBC systems is understanding and modeling the human body channel. Early research [2,3,4,5,6,7,8,9,10] focused on measuring the gain/attenuation profile for IBC, over the range of frequencies where the new application is believed to have the most efficient channel performance, which is from 100 kHz to 100 MHz, below which the periodic exposure to EM signals at certain power levels might have harmful health effects, and above 100 MHz the body antenna effects kicks in, leading to more transmitted power being leaked into the surrounding environment, rather than confined to the body.

In [11], the authors presented a simplified circuit model, that models the basic blocks of the IBC system. Taking into account different variables and parameters that explain the IBC channel behavior, geometrical and biological features of the human body in general and the body tissue’s in particular and the electrode contact impedance—which was studied and modeled in more details in [12] and finally the input /output impedances for the transmitter and receiver electrode. From the study and the model that followed, it became clear that the channel performance and characteristics slightly differ from one person to the other, due to the biological and body frame/geometrical features of each person. Such results paved the way for testing the feasibility of using IBC channel characteristics as a biometric identity for each person, due to the unique channel features that differ from one person to another, thus can be used for identification. In this paper, we introduce and test the feasibility and efficiency of using the IBC channel characteristics as a biometric identity.

Experimental measurements were carried out on five subjects and two five-layer physical phantoms prepared using tissue mimicking materials, then different machine learning algorithms were applied and used to train and test models for identifying and recognizing each of these subjects based on the features extracted from their IBC channel characteristics. The basic scientific question that this paper aims at tackling is to determine whether the characteristics of the intra-body communication channel compromise certain features that are unique for each individual, as well as testing the feasibility of using these features as biometrics for identifying such individual/personnel with high accuracy. The main contributions of this paper can be summarized as follows:

(1) Study and present a survey on prior work in using IBCs for biometric identity.
(2) Gather experimental data that characterizes the IBC channel characteristics and extract unique and identifying features.
(3) Applying machine learning algorithms on the collected data and features to use the IBC channel characteristics as a biometric identity.

The paper is organized as follows: in Section 2, a literature survey is presented and the basic concepts and features of IBC systems are introduced. In Section 3, the methodology and system are proposed, together with the experimental setup. Furthermore, the machine learning algorithms and approaches used for extracting the adequate features for subject identification are presented. In Section 4, the results obtained using these algorithms and models are reported and compared, and final recommendations for approaches usability, based on targeted applications, are presented. In Section 5, the paper’s findings are concluded.

## 2. Intra-Body Communications

### 2.1. Introduction

Intra-body communications can be categorized into two main types; capacitive coupling (near-field coupling method) and galvanic coupling [13,14,15,16,17,18]. In capacitive coupling, only the signal electrodes of the transmitter and the receiver are attached to the body while the ground (GND) electrodes are left floating in the air. The conductive body forms the forward path while the signal loop is closed through the capacitive return path between the transmitter and the receiver’s GND electrodes. The second approach, which depends on the galvanic coupling principle, uses a pair of electrodes for both the transmitter and the receiver to propagate the electromagnetic wave. The signal is applied over two coupler electrodes and received by two detector electrodes. The coupler establishes a modulated electrical field, which is sensed by the detector. Therefore, a signal transfer is established between the coupler and detector units by coupling minute signal currents into the human body. In both approaches, it has been shown that the attenuation of the body channel can be much lower than that of the air channel in frequencies up to 100 MHz [19]. An attractive feature of the galvanic coupling approach is that the signal is totally confined to the body, unlike capacitive coupling where the signal return path is established through the air. Galvanic coupled signals experience minimal interference from other electronic devices, enabling robust and secure data exchanges. For such reason, the galvanic approach is adopted by the authors as the method of choice for implementing the proposed system.

### 2.2. IBC as a Biometric Identity

In prior work [11], the authors proposed an accurate circuit model for the human arm as an IBC channel. In that model, the arm was simplified to the five main layers previously mentioned: muscle, fat, skin, cortical bone, and bone marrow. The model took into consideration system components, as the electrodes used, as well as biological factors, as the age and body mass. Since the gain profile obtained using the model showed very good match with experimental results previously reported in the literature, the authors adopted the model for the gain/attenuation calculations. Results based on such calculations show how the body’s behavior as a signal transmission channel depends on different features, both biological and geometrical, and is thus unique to each person. An advantage for using this proposed biometric over the conventional biometrics currently used, as the fingerprints, is that it can provide periodic and continuous identification/authorization with no required effort from the person and the two nodes can communicate for re-authorization periodically and seamlessly to the user.

The concept of IBC was first introduced, yet in a different form, in 2004 [20], through a device called Redtackon. The device was a prototype for a transceiver system that utilizes the Electrostatic (Capacitive) coupling IBC technology for communications, where the IBC channel acts as a medium for connecting the ID or the tool used for authentication with the other end where authentication is required, i.e., the body acts a safe communication channel for transporting the identity information, without the actual IBC cannel characteristics being used in the identification process [20,21,22]. In 2007, authors of [23] suggested using the IBC galvanic channel characteristics as a metric for identification among different subjects. Testing was performed on five subjects, where a technique based on Euclidian distance technique, calculated using the sum of the power spectrums at different frequency bands: 0–30 MHz, 30–60 MHz, and finally from 60 to 90 MHz was used to calculate the similarities between certain measured datasets and the different subjects to identify which subject it belongs to. The best verification rate of 58% was obtained in the 30–60 MHz sub-band. In [24], the same research group used the galvanic IBC channel response, but this time when the transmitter (TX) and receiver nodes (RX) are on the palm only. Support Vector Magnitude (SVM) machine learning technique was used, but still an identification error of 25% (verification accuracy of 75%) was achieved. In [25], authentication using biometric pulse response was introduced, where the subject under test holds brass cylindrical electrodes in his/her hand for signal flow, then a pulse is transmitted through the body, where the pulse response is recorded using an oscilloscope and is used for verification and authentication purposes. Although these trials did prove that IBC technology has the potential to be used as a biometric identity, verification accuracy results were not very encouraging, furthermore, the setup used, could not practically be used for daily life scenarios. In order to fill this gap, we proposed a simple yet effective method for using galvanic IBC channel response as a biometric identity for subject identification.

## 3. Proposed Method

The proposed system follows the methodology described below:

(1) An electrical signal is coupled into the body at the transmitter (TX) node, then picked up at the receiver nodes (RX), where the signal is transmitted through the body following the galvanic coupling IBC technique, since both electrodes at each node are connected to the body.
(2) The received signal incorporates information about the channel that it was transmitted through—the body in this case—which is unique for each person and can thus be used as a biometric identifier. This information is mainly based on the channel response (channel transfer function) which is determined mainly by the gain/attenuation channel profile that is developed due to the body’s unique biological and geometrical features, as discussed in [11].
(3) The above procedure would be repeated automatically *n* times for the calibration of the system, where *n* is a system parameter. Calibration is required to extract unique features to be able to accurately identify subjects, and to aid at normalizing the features against external factors whose effect should not impact the final unique biometrics, such as: temperature, wind, posture, illumination, etc.
(4) Once the system is calibrated the person’s identity will thus be stored, yet still will be unique and relevant to the specific system (hardware) used, adding an extra layer of security.
(5) Continuous authentication/identification/authorization can then take place, where a predefined signal is then transmitted from the TX node, every specific time interval. This is also one of the major advantages of adopting the proposed biometrics, where the identification/authentication process is done automatically and conveniently, with no need for any physical actions to be taken on the user’s part, as opposed to other techniques like using finger prints.
(6) The received signal is picked up at the RX node and further processed through machine learning algorithms to complete the identification process and verify the identity of the subject, after comparing the received signal with previously recorded identifiers that are unique for that subject.

### 3.1. Experimental Setup

**Figure 1:**
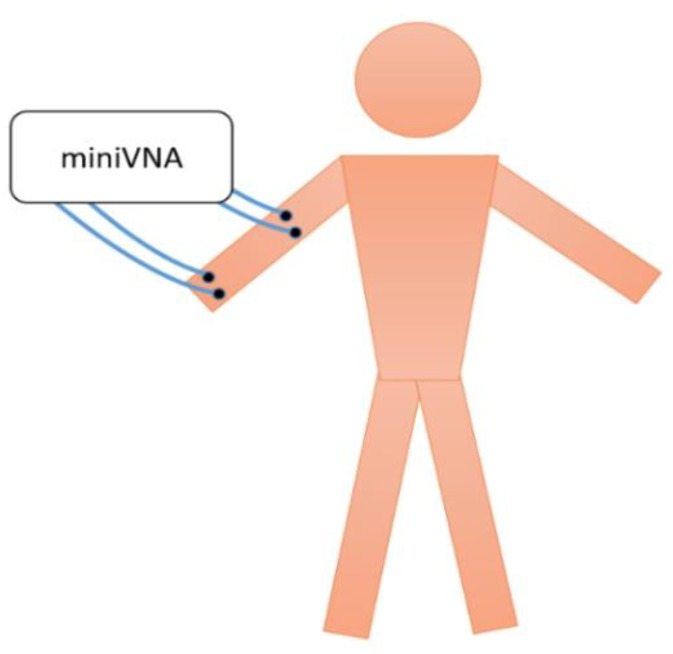
Experimental setup, where miniVNA (Vector Network Analyzer) is used to measure the channel gain/attenuation profile for different transmitter (TX)–receiver nodes (RX) separations and configurations.

**Figure 2:**
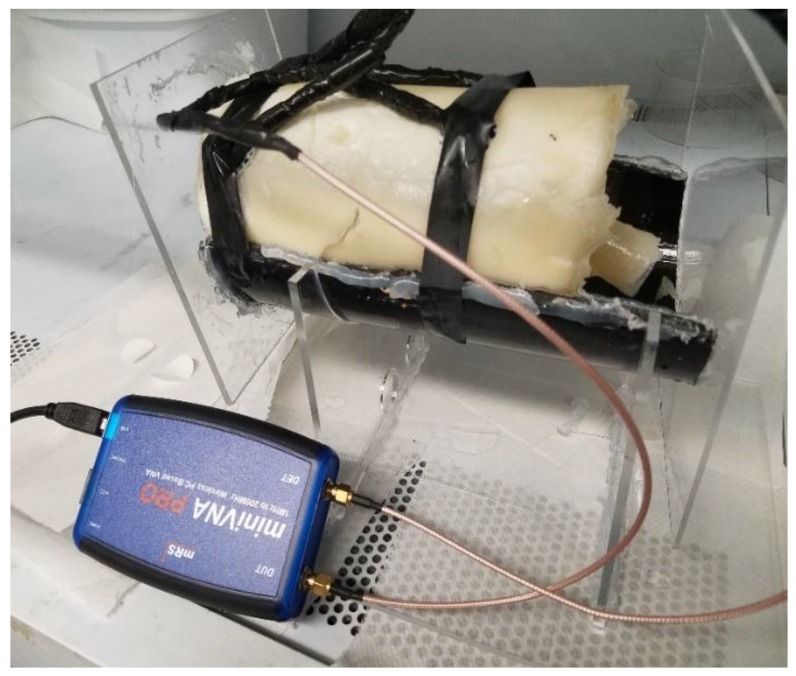
The constructed five-tissue layers arm phantom model in a measurement’s setup scenario (connected to the miniVNA).

**Figure 3:**
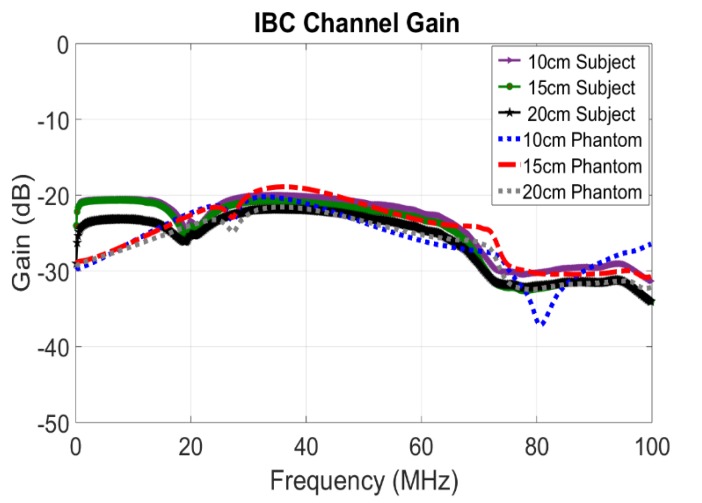
Intra-Body Communication (IBC) channel gain, for a human subject vs for a phantom, when varying the distance between the transmitter and the receiver to be at 10, 15, and 20 cm and separation between electrodes of each node is 5 cm.

Testing was performed using the miniVNA Pro [26], which is a handheld, portable Vector Network Analyzer that covers the frequency range of 100 kHz to 200 MHz. The miniVNA represents the TX/RX nodes that were discussed earlier, where a signal is transmitted at the TX node (port in this case) through the IBC galvanic approach, travels through the body and is shaped through the specific individual’s channel characteristics and profile, then picked up at the RX node (port). The test setup is shown in Figure 1, where the VNA was used to measure the S-parameters, especially S21, representing the channel gain. The maximum RF output power used was 0 dBm, thus adhering to the health safety limits [27,28]. Initially, the setup was tested on phantoms as shown in Figure 2, prior to human testing. Phantoms are physical five-layer model built using tissue mimicking materials that were developed by the authors in [29,30]. These materials accurately mimic the dielectric properties of the tissues; namely the permittivity and conductivity, which are mainly responsible for determining the transmission characteristics for the IBC channel. The five layers that the phantom model mimics are the skin, muscle, fat, cortical bone and bone marrow. Two phantoms were developed and used for testing. Same testing procedures were then performed on five real subjects (human beings), making the total number of tested subjects to seven distinct ones (two phantoms and five humans). Testing was done at room temperature, over dry skin. Different postures were tested, sitting and standing, yet no significant impact was observed, thus the remaining of the testing procedures were executed for the sitting posture. Measurements were carried out around noon time. Results obtained from testing on phantoms are precise and accurate enough to be used in comparison with results collected from real subjects, as shown in Figure 3, which further enhances the final results obtained from the developed machine learning models. Figure 3 shows measurements’ results for three scenarios where the distance between TX and RX were varied between 10, 15 and 20 cm, while the separation between electrodes of the same node are kept constant at 5 cm. More than 150 measurement instances were carried out, with 632 measurement point per instance.

To study the different features that can be used for subject identification, we explored various approaches:

(1) Using the amplitude of the galvanic channel gain at different frequencies directly as the features, over the whole frequency range, at each TX–RX separation distance separately. In other words, the channel gain/attenuation profile across the galvanic coupling frequency range is used directly to obtain the unique features determining the biometric identity of each individual, yet each set of these features are tested separately against each separation distance.
(2) Use the same approach as 1 but when using all the data for the different TX–RX separations, as different cases; as if different instances with the same unique identifiers (for example the channel response magnitude at 10 MHz is used as the same unique identifier, no matter what the separation is) thus increasing the number of instances that can be used for training and testing the machine learning models by 3× times.
(3) Same as approach 1, but when combining the measurements for all TX–RX separation measurements, not as test cases, but as features (channel response magnitude at 10 MHz for the 10 cm is used as a feature, while the magnitude at 10 MHZ for the 15 cm is used as a different feature, thus for each measurement case, the three separations are combined as a single training/testing instance). In other terms, features obtained from the channel profile when the separation distance for the experimental setup is 10 cm, are used all together with features generated in case of 15 cm and 20 cm at the same time to train and test the identification machine learning models, to check if adding such variations would enhance the model accuracy and performance, through adding extra unique and identifiable information.
(4) Divide the frequency spectrum into bins/segments of equal sizes (example: 1 MHz bin, 5 MHz bin), compute the total power spectral density for the channel response for the frequency components within each bin, then use this value as a single unique feature.

MATLAB [31] was used for signal processing, and to compute the mentioned features. These features were then imported to WEKA [32], a suite of machine learning software written in Java, developed at the University of Waikato, New Zealand under the GNU General Public License, for applying different machine learning algorithms for training and testing the data, and obtaining performance metrics for comparison between different approaches in feature selection as well as comparing different machine learning algorithms.

### 3.2. Machine Learning Algorithms

The machine learning algorithms used in this study are: Support Vector Magnitude (SVM), the k-nearest neighbor, J48, Random Forest and the Naïve Bayes classifier. The Support Vector Magnitude (SVM) is a discriminative classifier formally defined by a separating hyperplane. In other words, given labeled training data (supervised learning), the algorithm outputs an optimal hyperplane which categorizes new examples. In two dimensional space this hyperplane is a line dividing a plane in two parts where in each class lay in either side [33]. The k-nearest neighbors (KNN) is a non-parametric, lazy learning algorithm. Its purpose is to use a database in which the data points are separated into several classes to predict the classification of a new sample point. The J-48 is the WEKA implementation of the C4.5 algorithm which builds decision trees from a set of training data using the concept of information entropy. At each node of the tree, C4.5 chooses the attribute of the data that most effectively splits its set of samples into subsets enriched in one class or the other. The splitting criterion is the normalized information gain (difference in entropy). The attribute with the highest normalized information gain is chosen to make the decision. The C4.5 algorithm then recuses on the partitioned sub lists [34]. Naïve Bayes classifiers are a family of simple "probabilistic classifiers" based on applying Bayes’ theorem with strong (naive) independence assumptions between the features. Finally, Random Forest classifiers are an ensemble learning method for classification, regression and other tasks that operates by constructing a multitude of decision trees at training time and outputting the class that is the mode of the classes (classification) or mean prediction (regression) of the individual trees [35].

These machine learning algorithms are then used to train and test models that can be utilized for individuals’ identification using the various proposed features, extracted from the IBC channel characteristics, that were obtained through the measurements performed using the experimental setup explained. Different features, for each of the proposed methodologies, are then used to train the model to be able to recognize each individual according to his/her IBC characteristics’ features. The models are then tested against a testing set of instances, where the features are fed to the model and then the model should be able to identify the individual accurately and preciously. The basic performance metrics that will be used for comparison of the adopted machine learning algorithms are: accuracy, precision, recall and F-measure. Accuracy is simply the percentage of correctly predicted cases, among all the predicted ones; in other terms, how many times the algorithm was correct in its final prediction. Precision for a class is the number of true positives (i.e., the number of items correctly labeled as belonging to the positive class) divided by the total number of elements labeled as belonging to the positive class (i.e., the sum of true positives and false positives, which are items incorrectly labeled as belonging to the class). Recall is defined as the number of true positives divided by the total number of elements that actually belong to the positive class (i.e., the sum of true positives and false negatives, which are items which were not labeled as belonging to the positive class but should have been). F1-score combines both metrics, for getting a more accurate performance metric.

## 4. Results

In this section, results for testing the use of IBC channel characteristics as a biometric identity for identification applications, where the job of the biometric classifier is to determine to which user a certain measurement sample belongs to, are presented. The basic performance metrics, namely, the accuracy, precision, recall, and F-measure are shown in Table 1, Table 2, Table 3, Table 4, Table 5 and Table 6, for the different machine learning algorithms adopted in this study; Naïve Bayes, SVM, KNN, Random Forest, and J48. Results are shown for the four different approaches followed for choosing the adequate features that would not complicate the model too much (not adding extra unnecessary features, to save computational time and power), yet preserving the model’s accuracy and performance in doing its main task—identifying the individual according to his/her IBC channel characteristics’ biometrics. In Table 1, Table 2 and Table 3, results for applying different machine learning algorithms, using the WEKA software, are shown, where the standard performance metrics are reported, for the results when the first approach (the gain at each frequency component is used as a feature), for each of the TX–RX separations of 10, 15, and 20 cm. Total number of features used for each case is 632 feature, and a cross validation of 10-fold technique is used for training/testing. As can be seen from the results, and is expected as well, when using measurements obtained from the 10 cm separation, results are better, reaching an identification accuracy of 98%–100%. Yet the accuracy drops, for the same classifiers, when using data for larger separations (15 and 20 cm). Once again, such findings are expected, since the signal suffers less attenuation for smaller separation between the communicating nodes, yet as the separation increases, the attenuation increases. For approach two, results from all TX–RX (Transmitter–Receiver) separations are used in a single training/test run, meaning that test trials are all used as train/test cases, yet each is considered as a separate case. Total number of features per case stays the same, at 632. In this case, the system is trained using the 10 and 15 cm cases and is tested on the 20 cm cases. Results are shown in Table 4. In spite of the fact that the characteristics and geometry changes, the performance reaches an accuracy of 89% for the KNN classifier, which is still an acceptable result, given that training and testing are performed for different TX–RX separation cases. However, such drop in accuracy is also expected. As shown from results presented in Table 1, Table 2, and Table 3, features extracted from the channel characteristics are more powerful in identifying a certain individual (more unique and distinguishable) when the separation distance is less (10 cm yields the best results in this case), since the channel attenuation increases with distance, thus the received signal power is reduced significantly with increasing the separation between the TX and RX nodes.

This explains why approach 2 yields worse results than approach 1 (in the 10 cm separation case), where extra training and testing cases are added yet with less powerful features, which caused more confusion to the models when trying to estimate the best features to uniquely identify an individual from the proposed features, thus the overall models’ accuracy and ability to precisely identify the individual’s identity were slightly reduced. In approach three, each frequency component has three different features (gain at 10, 15, and 20 cm). Results for this case are shown in Table 5 where the performance stays at a very-good level of ~98%, since more features are added to the system. The SVM classifier did not converge for both cases. Slight improvements in some of the models’ performance (for the Random Forest and the J48 algorithms) are observed, yet at the cost of extra complexity, where the number of features are increased by 3×, which means the models’ complexity is increased, more computing resources are needed and more memory for storing the model parameters is also needed, which might not be the best choice then for most of the IBC and wearables’ applications, if such systems are tight on power and area budgets. The final approach attempted was to divide the spectrum into power bins of equal sizes (example: 1 MHz bin and 5 MHz bin), compute the total power for the frequency components within each bin, then use it a single feature. This approach is more resilient to noise and system/environment changes, as it computes the feature as the average power within a range of frequencies, not just at a single relative one, thus has higher ability to eliminate noise and irregular changes. In Table 6, we show the performance metrics for the case when the spectrum is divided into bins of size 0.5 MHz each. The performance shown in Table 6 is superior to other approaches and hence will be adopted as the method of choice.

### Impact of Bin Size

**Figure 4:**
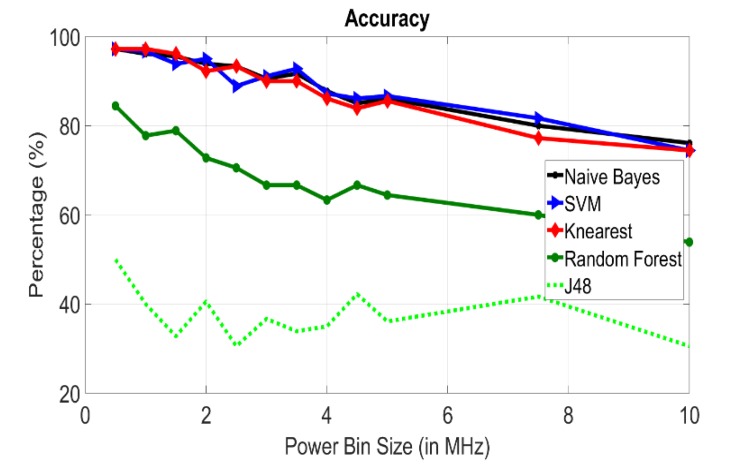
Accuracy for different classifiers versus power bin size.

**Figure 5:**
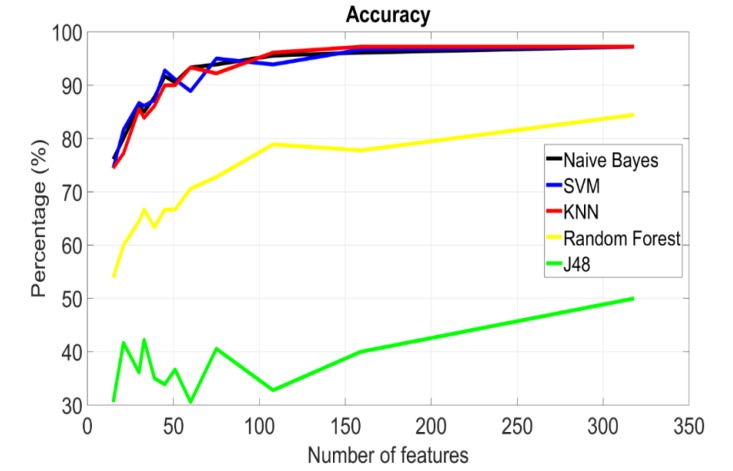
Accuracy for different classifiers versus the number of features.

This section investigates the performance of the power binning approach versus the size of the power bin. By referring back to Figure 3, one can see that the gain drops for frequencies over 50 MHz. This occurs due to different reasons; body antenna effect, causing power leakage as well as signal interference, and the attenuation introduced by body tissues. This is also confirmed in [23], where the frequency band of less than 60 MHz showed better results. Thus, to reduce the number of features, we consider frequencies between 100 KHz and 50 MHz. Results are plotted in Figure 4 showing the tradeoff between accuracy and bin size where the identification accuracy drops as the power bin size increases (as the number of features decreases). Figure 5 shows the tradeoff between the accuracy and the number of features, where accuracy improves significantly with increasing the number of features then saturates beyond a certain value, after which adding more features does not contribute much to the identification process. From the findings we concluded that the averaged power bin approach is best, with bin size of 0.5 MHz, over the frequency range of 100 KHz to 50 MHz, with either the KNN or Naïve Bayes classifiers.

The results show that features extracted from the IBC channel characteristics can be accurately used as identity biometrics. However, which approach to be used in selecting the features, as well as, which machine learning algorithm to be picked, will depend on the application at hand. If the performance metrics of the machine learning models, such as the accuracy or precision, are the main concern, then both the first and fourth approaches can be used for selecting the right features, with the Naïve Bayes or the KNN algorithms as the algorithm of choice. Approach 1 (for the 10 cm separation case) is much simpler than approach four, since minimal computation is needed for feature preprocessing, as the channel response at different frequency instances are directly used as the features. While, in approach four, extra steps are needed to compute the power spectral density for each bin. However, approach four shows better resilience to changes in external conditions and biological variations such as moisture and other factors. Approach two showed worse overall performance results, plus it needs extra experimental configuration (different separation distances), thus it is not a preferred approach. Finally, approach three shows slight improvement for the random forest and J48 algorithms, yet at a much higher computational cost, thus not a very attractive approach for wearables and ultralow power biomedical applications. To summarize, approach 1 for features selection while using the Naïve Bayes or the KNN model, provides good results, with the simplest model and least computational power budget. Approach four with the Naïve Bayes model provides good results as well, and is more resilient to noise and variations in external factors, yet requires extra computational steps, thus, a higher power budget. The choice thus depends on the application at hand: the computational budget versus model accuracy and resiliency. Findings are summarized in Table 7.

## 5. Conclusions

In this paper, the characteristics of the Intra-Body Communications channel, derived from the channels’ gain/attenuation profile, is used as a biometric identity for subject identification. In addition to five real subjects (humans), two multi-layer physical phantom models are introduced and used as well, to diversify and further validates the feasibility of the study. Channel characteristics obtained from the experimental measurements collected are further processed for feature extraction, where different approaches were compared to determine the most unique and distinguishable features for the channel characteristics. Using frequency bins of 0.5 MHz wide each, and covering the frequency spectrum from 100 kHz to 50 MHz, the extracted features were then used to compare between the accuracy and efficiency of different machine learning algorithms, where tradeoffs between accuracy, number of features, and computing resources were studied and presented as well. From the study, the best performance was obtained using the Naïve Bayes algorithm, with an identification accuracy of 98.5% and precision and recall of 0.984 and 0.984, respectively. The k-nearest neighbors (KNN) yielded the second best results, with identification accuracy of 97.8% and precision and recall of 0.978 and 0.978, respectively. From the results, it is proven that the IBC channel characteristics can be accurately used as a biometric identity, where a simple and portable setup can be used for such purpose. Such technology can be used for security applications in general, and as security layer for body area networks specifically, for securing data transmission between different nodes and sensors in or on the body. Future work will focus on testing for more IBC channel related features as well as testing the use of the IBC channel features for biometric authentication systems.

## Figures and Tables

**Table 1:** Performance metrics for TX–RX (Transmitter–Receiver) of 10 cm features (632 features per case).

Classifier	Accuracy	Precision	Recall	F-Measure
**Naïve Bayes**	98.8372%	0.989	0.988	0.988
**SVM**	95.5%	0.962	0.955	0.955
**KNN**	100%	1	1	1
**Random Forest**	96%	0.962	0.960	0.959
**J48**	92%	0.939	0.920	0.923

**Table 2:** Performance metrics for TX–RX of 15 cm features (632 features per case).

Classifier	Accuracy	Precision	Recall	F-Measure
**Naïve Bayes**	91.9811%	0.920	0.920	0.920
**SVM**	90.566%	0.909	0.906	0.907
**KNN**	91.9811%	0.921	0.920	0.920
**Random Forest**	91.9811%	0.921	0.920	0.920
**J48**	81.6038%	0.827	0.816	0.818

**Table 3:** Performance metrics for TX–RX of 20 cm features (632 features per case).

Classifier	Accuracy	Precision	Recall	F-Measure
**Naïve Bayes**	71.6102%	0.740	0.716	0.691
**SVM**	80.5085%	0.838	0.805	0.812
**KNN**	83.8983%	0.750	0.581	0.655
**Random Forest**	79.661%	0.793	0.797	0.782
**J48**	78.3898%	0.606	0.645	0.625

**Table 4:** Performance metrics for all TX–RX as different cases (632 features per case).

Classifier	Accuracy	Precision	Recall	F-Masure
**Naïve Bayes**	85.9756%	0.844	0.609	0.708
**SVM**	---	----	----	-----
**KNN**	89.4817%	0.894	0.895	0.894
**Random Forest**	87.0427%	0.870	0.870	0.868
**J48**	82.3171%	0.833	0.823	0.826

**Table 5:** Performance metrics for all TX–RX as different cases (1896 features per case).

Classifier	Accuracy	Precision	Recall	F-Measure
**Naïve Bayes**	97.9695%	0.981	0.980	0.980
**SVM**	----	-	-	-
**KNN**	96.9543%	0.970	0.970	0.970
**Random Forest**	97.9695%	0.981	0.980	0.980
**J48**	92.3858%	0.941	0.924	0.926

**Table 6:** Performance metrics for the power bin approach (0.5 MHz bin size).

Classifier	Accuracy	Precision	Recall	F-Measure
**Naïve Bayes**	98.4127%	0.984	0.984	0.984
**SVM**	94.9206%	0.963	0.949	0.952
**KNN**	97.7778%	0.978	0.978	0.978
**Random Forest**	87.619%	0.892	0.876	0.863
**J48**	47.9365%	0.750	0.479	0.512

**Table 7:** Summary for the different features selection approaches and best performance result for each.

	Description	Recommended Model and Performance	Comments
**Approach 1**	Using the magnitude of channel response at different frequencies as features, considering only the best separation distance	Naïve Bayes (98.8372%) KNN (~100%)	- Achieves high accuracy with simple models, in case of the 10 cm separation.
**Approach 2**	Same as approach 1, but all the separation distances are used as different training/test cases for the same run (number of train/test cases are 3× that of approach 1)	KNN (89.4817%) Random Forest (87.427%)	- Simple models, yet least accuracy among all approaches
**Approach 3**	Same as approach one, but channel response at different frequencies and separation distances are all combined as features (3× number of features compared to approach 1 and 2)	Naïve Bayes (97.9695%) Random Forest (97.9695%)	- More complex models (3× number of features) and more computational resources needed.
**Approach 4**	Use of power Bins as features (integration of power across a frequency spectrum (bin))	Naïve Bayes (98.4127%) Random Forest (97.7778%)	- High accuracy - More resilient to noise and magnitude variations - Extra features’ pre-processing is needed
